# Preparation and characterization of edible films from gelatin and hydroxypropyl methyl cellulose/sodium carboxymethyl cellulose

**DOI:** 10.1016/j.heliyon.2024.e41613

**Published:** 2024-12-31

**Authors:** Jingjing Du, Qian Zhu, Jiagang Guo, Jiayu Gu, Jianlong Guo, Yuhan Wu, Ling Ren, Song Yang, Jian Jiang

**Affiliations:** a Institute of Agro-products Processing, Anhui Academy of Agricultural Sciences, Hefei, Anhui, 230041, China; b Anhui Engineering Laboratory for Functional Microorganisms and Fermented Foods, Anhui Academy of Agricultural Sciences, Hefei, Anhui, 230041, China; c Food Engineering College, Anhui Science and Technology University, Fengyang, 233100, China; d Huaibei Shunfa Food Co., Ltd, No.10 Qianlong Avenue, Huaibei, Anhui, 235100, China

**Keywords:** Gelatin, Hydroxypropyl methylcellulose, Carboxymethyl cellulose

## Abstract

This study investigates the enhancement of gelatin (GEL) films using hydroxypropyl methylcellulose (HPMC) and carboxymethyl cellulose (CMC) for edible film packaging applications. Although GEL is biocompatible and cost-effective, its limited mechanical strength presents significant challenges for practical applications. The findings indicate that CMC effectively increases tensile strength (TS), while HPMC improves elongation at break (EAB) and hydrophilicity. Notably, the composite modification with HPMC and CMC proves to be more effective than individual modifications. The optimal HPMC/CMC ratio of 3:7 resulted in blend films that exhibited the highest TS and lowest water vapor permeability (WVP). Fourier transform infrared (FTIR) analysis revealed enhanced hydrogen bonding between the polymers, while scanning electron microscopy (SEM) and thermogravimetric analysis (TGA) confirmed a more uniform structure with improved thermal stability in the blend films. These results suggest that optimizing the HPMC/CMC ratio in gelatin-based films can significantly enhance their mechanical, barrier, and thermal properties, providing new possibilities for their application as food packaging materials.

## Highlights

• HPMC improved the water solubility of the blend films, CMC had the opposite effect.
• The appropriate concentration ratio of HPMC and CMC is crucial for improving the properties of the GEL-based film.
• The 3:7 HPMC/CMC ratio enhanced tensile strength, water vapor permeability, and thermal stability, surpassing individual modifications.

## Graphical abstract

**Figure:**
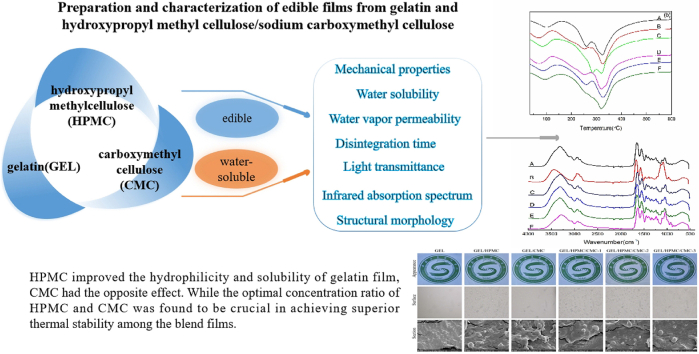

## 1 Introduction

The increasing accumulation of non-biodegradable materials has significant detrimental effects on the environment. Consequently, there is an urgent need to develop biodegradable films derived from biopolymers to mitigate the adverse effects associated with non-biodegradable waste [1,2]. The main components for producing edible/biodegradable films include proteins, carbohydrates, solubilizing medium, and plasticizer [3]. Edible and water-soluble food packaging films made from natural macromolecular polymers represent an important type. Gelatin (GEL) is a naturally occurring biopolymer derived from the hydrolysis of collagen, a protein found in the connective tissues of animals [4]. GEL is one of the most widely used biopolymers in food packaging films [5]. It has several advantages, such as low cost, biocompatibility, biodegradability, and film-forming properties [[6], [7], [8], [9]]. However, the proportion of hydrophilic amino acids in GEL is relatively high, and its water absorption capacity is high [10]. As a result, gelatin-based films were prone to expansion or rupture after absorbing water. Meanwhile, the poor mechanical strength of gelatin-based films limits their applications in the field of food packaging [11]. The physical and mechanical properties of gelatin-based films depend on the raw material source, extraction method, plasticizers, fillers, and cross-linkers. Therefore, researchers typically perform physical, chemical, and enzymatic modifications on GEL to enhance its applicability for food packaging [12]. Various natural and synthetic materials are often blended with GEL to improve its packaging properties [13,14]. At present, natural polymers blended with GEL mainly include polysaccharides, proteins, cellulose and its derivatives [[15], [16], [17]]. Hydroxypropyl methyl cellulose (HPMC) and carboxymethyl cellulose (CMC) possess non-toxic, odorless, and cost-effective characteristics, ensuring their safety and reliability. Both exhibit excellent biocompatibility, along with notable film-forming capabilities. Consequently, they find widespread application in both the pharmaceutical and food industries [18,19]. Furthermore, numerous studies have consistently demonstrated that the inclusion of either HPMC or CMC enhances the physicochemical and mechanical attributes of gelatin-based films. HPMC, a non-ionic cellulose derivative, possesses partially O-methylated and O-(2-hydroxypropylated) moieties [20]. Research has shown that the addition of HPMC resulted in a decrease in the enthalpy of fusion and the melting temperature of GEL films. However, it also led to an increase in the elongation, hydrophilicity, and in vitro bio-adhesion force of these films [21]. Additionally, the high hydrophilicity and water solubility of HPMC contribute to its excellent processability and ease of treatment during film formation. The uniform and stable dispersion formed by HPMC in water simplifies the casting and drying processes. Carboxymethyl cellulose (CMC) arises from the carboxymethylation of cellulose, a chemical process that converts the hydroxyl groups of cellulose into carboxymethyl groups [22]. The research demonstrated that the interaction between CMC and GEL molecules resulted in the formation of an interpenetrating network structure, which subsequently increased the tensile strength of gelatin-based film [23].

In this study, the mechanical properties, including tensile strength (TS) and elongation at break (EAB), as well as physical attributes such as water solubility, water vapor permeability (WVP), disintegration time, and light transmittance of gelatin-based films modified with HPMC and CMC, were systematically analyzed. Additionally, the microstructure, chemical structure, and thermomechanical properties of modified GEL films were investigated. The objective of this study was to assess the viability of GEL/HPMC/CMC blend films for use in edible and water-soluble food packaging applications.

## 2 Materials and methods

### 2.1 Materials

Gelatin powder (GEL, 250, food grade, Henan Xuri Group Co., Ltd, China). Hydroxypropyl methyl cellulose (HPMC), with a methoxyl group content ranging from 27.0 % to 30.0 % and a hydroxypropyl content between 4.0 % and 7.5 % (Beijing Hercules Co., Ltd., China). Carboxymethyl cellulose (CMC), with a pKa value of 3.5 and a molecular weight (Mv) of 90,000 (Henan Xuri Group Co., Ltd., China). Analytical-grade laboratory chemicals were acquired from National Pharmaceutical Group Co., Ltd., China.

### 2.2 Film preparation

A quantity of GEL powder weighing 3.0 g was dissolved in 100 mL of distilled water. This dissolution process was conducted at a temperature of 70 °C with continuous stirring at 400 rpm for 30 min. After complete dissolution, 0.5 g of glycerol was added. HPMC and CMC were added to the prepared solution in various proportions (HPMC:CMC = 10:0, 7:3, 1:1, 3:7, 0:10, w/w; total solid weight of 0.18 g), resulting in blends named GEL/HPMC, GEL/HPMC/CMC-1, GEL/HPMC/CMC-2, GEL/HPMC/CMC-3, and GEL/CMC. The solution was stirred for an additional 30 min at a constant temperature of 70 °C. Subsequently, the film-forming solution (50 mL) was poured into petri dishes and allowed to dry at 40 °C for 24 h. The GEL film served as the control group, excluding HPMC and CMC. he films were gently removed from the petri dishes and equilibrated at a relative humidity (RH) of 65 % and 25 °C for 7 days prior to undergoing measurements.

### 2.3 Film characterization

#### 2.3.1 Thickness and moisture content

The thickness of the films was measured using a digital micrometer (DL91150, precision 0.001 mm, Dongguan, China) at five randomly selected points on the film surface [24]. The films were cut into 2.5 cm × 2.5 cm squares and weighed before and after drying in an oven at 40 °C for approximately 12 h. The moisture content of the films was determined according to the method of Zafar et al. [25].

#### 2.3.2 Mechanical properties

The mechanical properties of films were evaluated according to method described by Souza et al. [26]. An intelligent electronic tensile testing machine (XLW/EC, Labthink Instruments Co., Ltd., Jinan, China) was used. The films were cut to dimensions of 100 mm × 15 mm, with an initial clamping distance of 60 mm, and tested at a speed of 50 mm/min.

#### 2.3.3 Solubility

Water solubility(WS) was determined according to Stevenson et al. [27]. The film samples were cut into 20 mm × 20 mm pieces and dried to a constant weight (*W*_*1*_). The dried film was placed in a sealed Petri dish with 20 mL distilled water at 25 °C for 24 h. Afterward, the insoluble film was dried again to a constant weight (*W*_*2*_). Water solubility was calculated using the following equation (1).

(1) $$WS\;\left(\%\right)=\frac{W_{1}-W_{2}}{W_{1}}\times 100$$

#### 2.3.4 Water vapor permeability (WVP)

The water vapor transmission of the films was measured using a WVP tester (W3/060, Labthink Co., Ltd, China) following the experimental approach advocated by Zheng et al. [28]. The samples were cut circles, each with a diameter of 80 mm, and securely affixed to the lid of a test cup containing 10 mL of distilled water. The gravimetric method was employed to measure the samples under controlled conditions of 38 °C and 70 % RH. The weight variations in the samples were monitored until a constant weight was attained.

#### 2.3.5 Disintegration time

The disintegration time was measured following the method of Tedesco et al. [18]. A droplet of phosphate-buffered saline solution (pH 6.75) was applied to the surface of the film (2 cm × 3 cm) at 37 °C. The time required for complete dissolution of the droplet, resulting in pore formation, was recorded.

#### 2.3.6 Determination of light transmittance

The films were precisely trimmed to dimensions of 10 mm × 40 mm at 65 % RH and 25 °C. Subsequently, the light transmittance of the films was measured using a UV spectrophotometer (V-5800, Metash Instruments Co., Ltd, Shanghai, China) over a wavelength range from 200 nm to 800 nm [29]. The empty quartz cuvette was used as the control.

#### 2.3.7 Fourier transform infrared (FTIR) spectroscopy

The infrared spectral analysis was conducted utilizing a Fourier Transform Infrared (FTIR) spectrometer (PerkinElmer, Waltham, MA, USA). The spectral range of 4000 to 400 cm^−1^ was comprehensively analyzed, entirely at room temperature [30]. The film was meticulously cut into a circular shape with a diameter of 15 mm. Each film was scanned 64 times. The air spectrum was used for background correction.

#### 2.3.8 Morphological and structural characterization

An optical microscope was used to observe the surface structure of the films at 40 × magnification. The cross-sectional structure of the films was inspected using a scanning electron microscope (Phenom XL, Netherlands) to analyze their morphology. The cross-sections were secured with conductive adhesive paper, and the films were sputtered with gold for 60 s to enhance conductivity [31].

#### 2.3.9 Thermogravimetric (TG) analysis

A sample (8.0 g) was placed in a crucible and heated from 25 °C at a rate of 20 °C/min under a nitrogen atmosphere. The method was adapted fromYan et al. [32].

### 2.4 Statistical analysis

Statistical analyses presented in this study were conducted using the SPSS software (IBM, USA) and Origin 9.0 (OriginLab, USA).

## 3 Results

### 3.1 Thickness, moisture content and mechanical properties

The moisture content of GEL film was approximately 6.95 %, as shown in Table 1. The blend films exhibited higher moisture content compared to GEL film, due to a greater number of hydroxyl and carboxyl groups. This enhanced hydrophilicity allowed the blends to absorb more moisture. HPMC had more hydroxyl groups than CMC; thus, the impact of HPMC on increasing the moisture content of gelatin-based films was significantly greater than that of CMC. The moisture content of GEL affected overall film performance, including properties such as tensile strength (TS, MPa) and elongation at break (EAB, %). GEL proteins were abundant in non-ionized polar amino acids, which facilitated the formation of numerous hydrogen bonds among the proteins and subsequently elevated the cohesion strength of the gelatin-based film [33]. The addition of HPMC resulted in a decrease in the TS of the film to 4.37 MPa, which was due to HPMC disrupting the continuity of the GEL matrix and leading to the formation of more deformable, random molecular structures. This increased flexibility allowed the film to undergo greater deformation before breaking, enhancing its ductility. As shown in Table 1, the EAB of the GEL/HPMC film rose to 166.90 %. In contrast, the TS and EAB of the GEL/CMC composite were measured at 14.34 MPa and 126.12 %, respectively. The addition of CMC enhanced the TS of the film while concurrently reduced its EAB. This was because CMC, with its linear polymer structure and higher rigidity, reinforced the GEL matrix by improving intermolecular interactions, leading to greater cohesion and strength. However, the increased rigidity reduced the film's flexibility, resulting in a decrease in EAB. As shown in Table 1, the TS of the GEL/HPMC/CMC-3 film (16.39 MPa) was the highest among all films. This indicated that an appropriate CMC concentration could effectively compensate for the excessive aggregation and uneven distribution of HPMC in the blend film.

**Table 1:** Thickness, moisture and mechanical properties of the films.

Samples	Thickness/nm	Moisture content/%	TS/MPa	EAB/%
GEL	0.17 ± 0.01	6.95 ± 0.82	5.54 ± 0.81	117.09 ± 4.56
GEL/HPMC	0.19 ± 0.02	11.71 ± 1.01	4.37 ± 0.35	166.90 ± 6.84
GEL/CMC	0.19 ± 0.01	7.02 ± 1.13	14.34 ± 2.01	126.12 ± 5.12
GEL/HPMC/CMC-1	0.19 ± 0.03	8.37 ± 0.94	7.27 ± 0.93	152.57 ± 0.01
GEL/HPMC/CMC-2	0.19 ± 0.01	7.57 ± 0.87	13.96 ± 0.92	140.80 ± 4.81
GEL/HPMC/CMC-3	0.19 ± 0.01	7.19 ± 0.64^d\^	16.39 ± 0.58	135.63 ± 5.67

### 3.2 Water solubility

When the films were submerged in water, the hydrogen bonding among various polymeric chains dissociated due to the competitive interaction with water molecules, leading to the decomposition and dissolution of the films [34]. Table 2 showed the water solubility of films measured at 25 °C. The solubility of the GEL/HPMC film increased significantly, reaching 70.11 %. The water solubility of GEL/HPMC/CMC-1, GEL/HPMC/CMC-2 and GEL/HPMC/CMC-3 was 65.92 %, 58.67 % and 55.60 %, respectively. This indicated a positive correlation between water solubility and HPMC concentration. The hydrophilic nature of HPMC, when combined with GEL, enhanced the swelling and dissolution of the film, leading to increased solubility. On the other hand, the solubility of GEL/CMC film decreased to 53.04 %. When CMC was combined with GEL, the interaction between the two polymers led to the creation of a more stable and less soluble film structure. Additionally, the presence of carboxyl groups in the CMC contributed to the decrease in solubility by forming hydrogen bonds with water molecules, thereby slowing down the dissolution process of the blend films.

**Table 2:** Water solubility, water vapor permeability (WVP) and disintegration time of the films.

Samples	Water solubility(%)	WVP × 10^-11^ g m^−1^·s^−1^·Pa^−1^	Disintegration time (s)
GEL	55.38 ± 3.75	2.38 ± 0.07	16.31 ± 1.06
GEL/HPMC	70.11 ± 2.79	2.86 ± 0.08	15.25 ± 1.33
GEL/CMC	53.04 ± 5.91	2.40 ± 0.04	16.59 ± 1.19
GEL/HPMC/CMC-1	65.92 ± 2.75	2.39 ± 0.08	16.81 ± 0.47
GEL/HPMC/CMC -2	58.67 ± 3.50	2.37 ± 0.03	17.89 ± 0.59
GEL/HPMC/CMC-3	55.60 ± 4.74	2.30 ± 0.07	18.87 ± 0.42

### 3.3 Water vapor permeability (WVP)

The water vapor permeability (WVP) was a vital parameter for determining the suitability of packaging materials in diverse applications [35]. As shown in Table 2, the WVP values of GEL film and GEL/HPMC film were 2.38 and 2.86, respectively. The high hydrophilicity of GEL/HPMC was due to the longer hydrophilic chains of HPMC, which were rich in hydroxyl groups and had a strong affinity for water molecules. These hydrogen bonds between the hydroxyl groups of HPMC and the water molecules acted as bridges, effectively linking the GEL chains with water. Consequently, the WVP of the GEL/HPMC film was significantly increased. The addition of CMC resulted in a slight increase in the WVP of GEL/CMC film. This was attributed to the larger anionic groups of the CMC, which increase free volume of the blend films. These anionic groups occupied more space within the blend films, creating additional voids or free volume within the GEL matrix. As shown in Table 3, the WVP of GEL/HPMC/CMC-1, GEL/HPMC/CMC-2 and GEL/HPMC/CMC-3 films was 2.39, 2.37, and 2.30, respectively. GEL, HPMC, and CMC interpenetrated, entangled, and interacted with each other through hydrogen bonding. CMC contained carboxyl and hydroxyl groups that formed hydrogen bonds with GEL and HPMC, creating a tighter, more interconnected network. This resulted in a cohesive film structure, reducing the mobility of water molecules and limiting the diffusion of water vapor, thereby decreasing WVP.

**Table 3:** Light transmittance of the films

Samples	Light transmission/%					
	350 nm	400 nm	500 nm	600 nm	700 nm	800 nm
GEL	62.47 ± 0.13	80.35 ± 0.89	87.36 ± 0.11	88.02 ± 0.14	89.06 ± 0.95	89.67 ± 0.82
GEL/HPMC	20.58 ± 0.54	40.12 ± 0.42	68.29 ± 0.59	74.07 ± 0.36	77.15 ± 0.17	77.92 ± 0.86
GEL/CMC	57.89 ± 0.47	65.96 ± 0.12	85.30 ± 0.62c	86.61 ± 0.82	87.14 ± 0.76	87.53 ± 0.39
GEL/HPMC/CMC-1	11.34 ± 0.24	18.68 ± 0.25	32.56 ± 0.16	38.73 ± 0.94	46.74 ± 0.47	59.34 ± 0.83
GEL/HPMC/CMC -2	25.39 ± 0.92	36.42 ± 0.33	52.56 ± 0.63	58.75 ± 0.48	64.10 ± 0.71	70.11 ± 0.36
GEL/HPMC/CMC-3	55.21 ± 0.14	64.12 ± 0.78	71.13 ± 0.35^c^	80.16 ± 0.37	81.41 ± 0.34	82.85 ± 0.96

### 3.4 Disintegration time

The disintegration time of GEL and GEL/HPMC films was 16.31 s and 15.25 s, respectively, as shown in Table 3. The addition of HPMC to the film formulation significantly decreased disintegration time of blend film. This finding indicated that phase separation occurred between GEL and HPMC, weakening the interaction between molecules. Consequently, the stability of the GEL/HPMC film was reduced, leading to a decrease in the disintegration time. The disintegration time of GEL/CMC film was 16.59s. A cross-linking reaction occurred between GEL and CMC, resulting in a slight decrease in solubility and an increased disintegration time. In contrast, the disintegration times of GEL/HPMC/CMC-1, GEL/HPMC/CMC-2 and GEL/HPMC/CMC-3 films were 18.61, 17.89 and 18.87, respectively. The differences in disintegration times among these blend films were mainly attributed to the close-packing nature of their internal structures, which significantly reduced the availability of free space and water channels during the disintegration process. The disintegration time of GEL/HPMC/CMC-3 was the longest, indicating that its structure was more compact. GEL and CMC formed the basic framework of the grid structure, while HPMC filled the grid pores. This combination led to the formation of a relatively regular and dense network, which slowed down the processes of water penetration and diffusion, thereby increasing the disintegration time.

### 3.5 Light transmittance

The light transmittance spectra of GEL, GEL/HPMC, GEL/CMC, GEL/HPMC/CMC-1, GEL/HPMC/CMC-2 and GEL/HPMC/CMC-3 films were presented in Table 3. In the visible light range of 350–800 nm, the transmittance of GEL film ranged from 62.47 % to 89.67 %. The decrease in light transmittance of the blend films was attributed to the phase separation effects that arose from the interactions between HPMC, CMC, and GEL. This phase separation led to the formation of an ordered network structure, which scattered light and reduced overall transmittance. However, the transparency of the blend films was positively correlated with the increasing concentration of CMC. The results demonstrated that adding CMC to the blend films effectively enhanced the compatibility of HPMC and GEL, leading to a more stable structure with fewer voids and defects, thereby improving structural integrity and facilitating light transmission.

### 3.6 Fourier transform infrared spectroscopy (FTIR) analysis

**Fig. 1:**
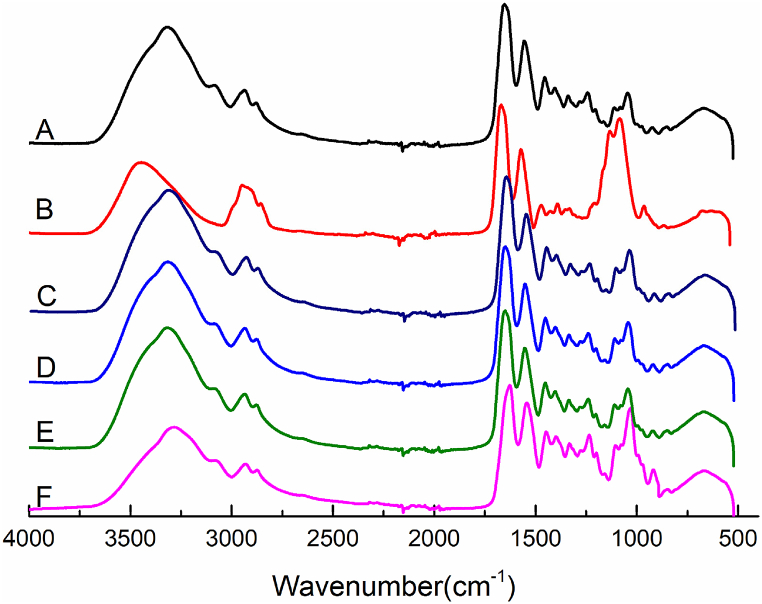
FTIR spectra for GEL (A), GEL/HPMC (B), GEL/CMC (C), GEL/HPMC/CMC-1 (D),GEL/HPMC/CMC -2 (E)and GEL/HPMC/CMC-3 (F) film.

Fig. 1 demonstrated that all films subjected to various blends exhibited absorption peaks characteristic of GEL film, with varying degrees of deviation. Table 4 showed the amide A band observed at 3308 cm^−1^ in the GEL film spectrum was attributed to the stretching vibrations of amine (N–H) and hydroxyl (O–H) groups. The amide I band, observed at 1653 cm^−1^, arose from the coupling of carbonyl (C=O) stretching vibrations with hydrogen bonding interactions involving the carboxyl (–COO). The amide II band, located at 1553 cm^−^^1^, was assigned to N–H bending vibrations coupled with C–N stretching of peptide bonds [36]. The amide III band, located at 1240 cm^−1^, corresponded to the C–N and N–H bonds within the bound amide structure. The spike at 1043 cm^−^^1^ was attributed to the vibrations of carbon-oxygen (C–O) and O–H [37]. In Table 4, a shift in the amide A band of the CEL/HPMC film from 3308 cm^−1^ to 3450 cm^−1^ was observed, indicating a potential electrostatic attraction between the two polymers. The amide I, amide II and amide III bands in the GEL/HPMC film shifted to 1670 cm^−1^, 1575 cm^−1^ and 1220 cm^−1^, respectively. HPMC contained a large number of –OH groups, while GEL contained polar groups such as–NH and –COOH, which could interact with each other through hydrogen bonds. These shifts suggested changes in the hydrogen bonding pattern due to the formation of new hydrogen bonds between GEL and HPMC.

**Table 4:** FTIR bands of the films.

Samples	Amide A (cm^−1^)	Amide I (cm^−1^)	Amide II (cm^−1^)	Amide III (cm^−1^)
	O–H stretching vibration	C=O stretching	Bending vibration N-H group, stretching vibration of C-N group.	Stretching vibration of C-N bands and N-H groups of bound amide, vibration of C-H groups of glycine
GEL	3308	1653	1553	1240
GEL/HPMC	3345	1670	1575	1220
GEL/CMC	3304	1639	1543	1228
GEL/HPMC/CMC-1	3324	1647	1548	1239
GEL/HPMC/CMC -2	3321	1645	1546	1237
GEL/HPMC/CMC-3	3288	1628	1542	1235

**Fig. 2:**
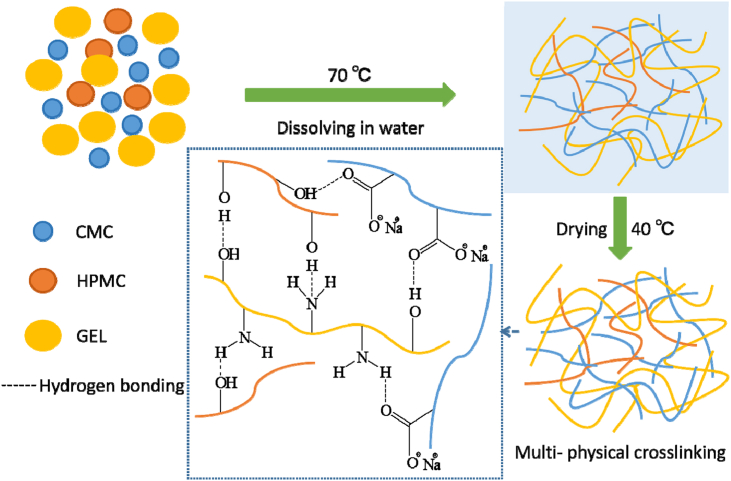
Multi-physical crosslinking of GEL/HPMC/CMC film.

The peak associated with O-H stretching vibrations in the GEL/CMC film (3304 cm^−1^) was similar to that of GEL film. However, the peak of amide I and amide II bands shifted to 1639 cm^−1^ and 1543 cm^−1^ in GEL/CMC film. This shift was attributed to changes in the secondary structure of the gelatin polypeptide chains induced by the addition of CMC. The amide II band corresponded to N–H bending vibrations coupled with C=O stretching of peptide bonds in gelatin [38]. The shift to higher frequencies suggested that the interaction between the –OH groups of CMC and the amino groups of GEL resulted in a more ordered and stable secondary structure. This was due to the formation of hydrogen bonds between the –OH groups of CMC and the amino groups of gelatin, leading to a more rigid structure [39]. Similarly, the amide III band, associated with C–N stretching and N–H bending, also shifted from 1240 cm^−1^ to 1228 cm^−1^, further corroborating the interaction between the –OH groups of CMC and the amino groups of gelatin during blending. Moreover, it was observed that the amide A band, amide I, amide II and amide III of CEL/HPMC/CMC-1, CEL/HPMC/CMC-2, and CEL/HPMC/CMC-3 film exhibited a shift towards lower wavelengths in Fig. 1. This shift suggested changes in hydrogen bonding patterns and N–H bending vibrations. The amide A band of CEL/HPMC/CMC-3 film shifted to 3288 cm^−1^, indicating that the hydrogen bonds interacting with the –OH groups in CEL/HPMC/CMC-3 were significantly weaker than those in the GEL film. In the GEL film, the –OH groups were relatively exposed, forming strong hydrogen bonds with water molecules [40,41]. In contrast, in the CEL/HPMC/CMC films, stronger and more complex hydrogen bonding occurred at specific concentration ratios, as illustrated in Fig. 2. This enhanced hydrogen bonding contributed to the observed peak shifts towards shorter wavelengths.

### 3.7 Structural morphology

**Fig. 3:**
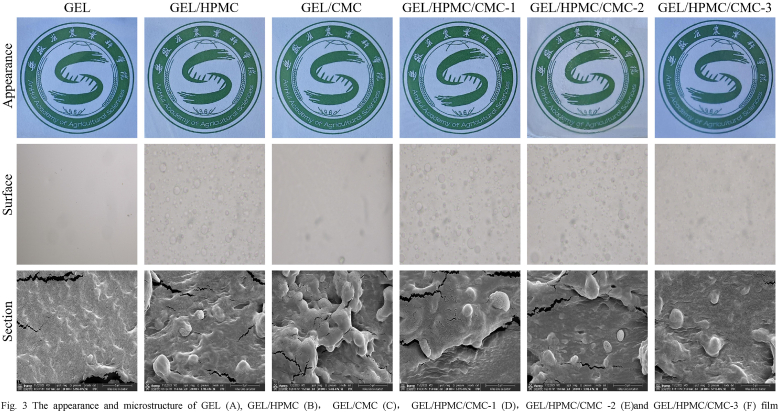
The appearance and microstructure of GEL (A), GEL/HPMC (B), GEL/CMC (C), GEL/HPMC/CMC-1 (D),GEL/HPMC/CMC -2 (E)and GEL/HPMC/CMC-3 (F) film.

The surface and cross-sectional morphologies of the films were presented in Fig. 3. The GEL film displayed a smooth and uniform structure, with no noticeable particles or imperfections. In contrast, particles were randomly distributed on the surface of the blend films, indicating partial incompatibility among GEL, HPMC, and CMC in the blended films. The cross-section of the GEL film was noticeably smoother than that of the blend films. Irregular protrusions and discontinuous regions were observed in the non-uniform cross section of blend films, indicating phase incompatibilities. The presence of HPMC or CMC hindered the formation of GEL junction regions, thus reducing the cohesion of the gel matrix. This resulted from differences in the polarity and chain lengths of the various polymers, leading to phase separation during film formation. The observed reduction in particulate protrusions and cracks in the GEL/HPMC/CMC-3 film (Fig. 3) was attributed to enhanced polymer compatibility. The optimized ratio of HPMC and CMC improved hydrogen bonding and minimized phase separation, resulting in a more uniform and cohesive film structure. This observation was consistent with the mechanical performance data, further substantiating the enhanced structural integrity of the film.

### 3.8 Thermal stability

**Fig. 4:**
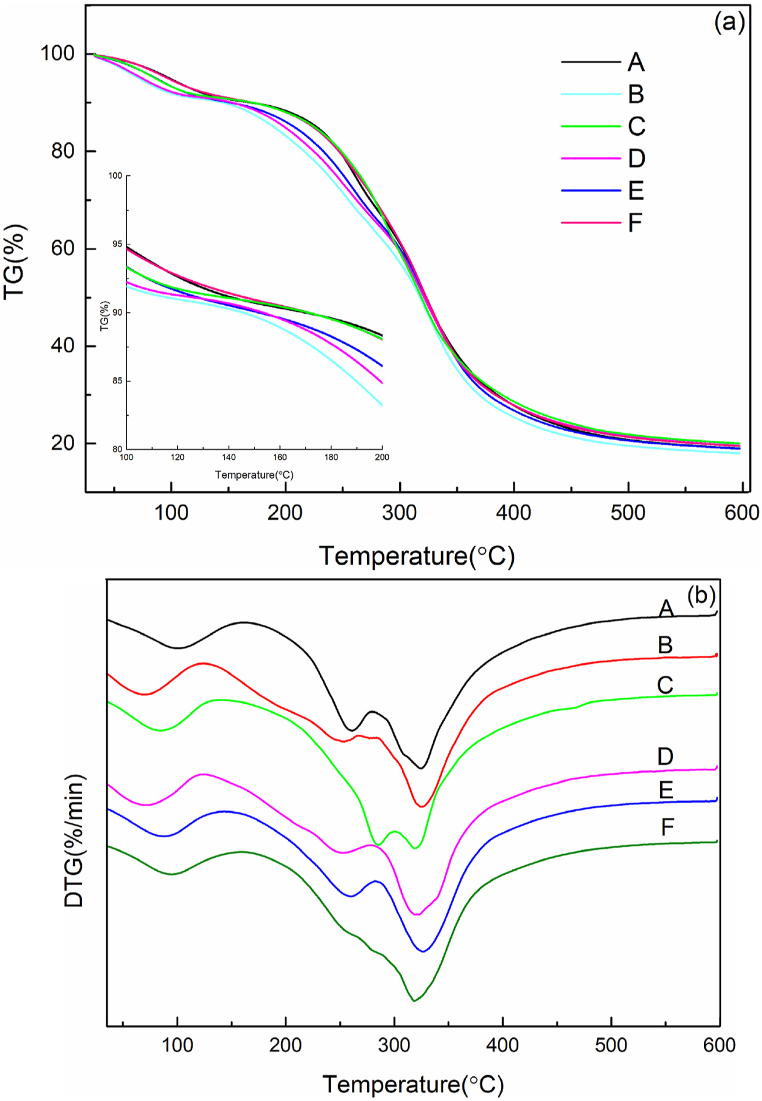
TGA (a) and DTG (b) images of GEL (A), GEL/HPMC (B), GEL/CMC (C), EL/HPMC/CMC-1 (D),GEL/HPMC/CMC -2 (E) and GEL/HPMC/CMC-3 (F) film.

The thermogravimetric analysis (TGA) curves for the films were shown in Fig. 4(a). All of the samples underwent two distinct stages during the thermal heating process. The first stage, below 250 °C, involved the loss of water, while the second stage corresponded to the thermal degradation of the polymer. The mass derivative curve (DTG) was categorized into three distinct stages, as shown in Fig. 4(b). In the first stage, the degradation rate was less than 20 % between 70 °C and 110 °C, mainly due to the evaporation of water in the film [42]. The degradation temperature in the second stage was primarily between 110 °C and 260 °C. The weight loss in this stage was primarily related to the decomposition of low molecular weight proteins of GEL [43]. The GEL/CMC film exhibited a peak at 284 °C in the second stage, as the low-molecular-weight GEL proteins interacted with CMC, leading to an increased degradation temperature. The thermal decomposition of GEL proteins occurred during the third stage, specifically within the temperature range of 260 °C–600 °C. The onset temperatures of the GEL, GEL/CMC, and GEL/HPMC/CMC-3 films were similar, ranging from 240 °C to 260 °C. The onset temperatures of the GEL/HPMC, GEL/HPMC/CMC-1, and GEL/HPMC/CMC-2 films ranged from 150 °C to 180 °C. The onset temperature of GEL/HPMC decreased to 160 °C. The excess HPMC significantly reduced the onset temperature of thermal degradation. This decline was attributed to the formation of agglomerates within the GEL matrix. These agglomerates disrupted the uniform structure of the matrix, creating weak points that compromised the film's integrity and diminished its thermal resistance, consistent with the SEM results. The GEL/HPMC/CMC-3 film exhibited only one peak around 320 °C, as shown in Fig. 4(b). This enhanced thermal stability was attributed to the appropriate concentrations of HPMC and CMC, which improved compatibility among the components within the GEL matrix. The different components formed a relatively uniform mixed system. Compatible components decomposed simultaneously or nearly simultaneously when heated, which was reflected in a relatively concentrated peak, indicating that this sample had higher thermal stability compared to the other samples.

## 4 Conclusions

The findings demonstrated that the composite modification with HPMC and CMC was more effective than individual modifications. The water vapor barrier properties and mechanical performance of the blend films were optimized by adjusting the HPMC/CMC ratio. Among the samples, the GEL/HPMC/CMC-3 film (with an HPMC/CMC ratio of 3:7) exhibited the highest tensile strength, the lowest water vapor permeability (WVP), and superior thermal stability. This improvement was attributed to the multi-physical crosslinking and optimized interactions between GEL, HPMC, and CMC, resulting in a more cohesive and uniform film structure. The optimized GEL/HPMC/CMC films showed strong potential as sustainable, edible, and water-soluble packaging materials, offering a promising eco-friendly alternative for food packaging applications.

## CRediT authorship contribution statement

**Jingjing Du:** Writing – original draft. **Qian Zhu:** Data curation. **Jiagang Guo:** Investigation. **Jiayu Gu:** Software. **Jianlong Guo:** Conceptualization. **Yuhan Wu:** Writing – review & editing. **Ling Ren:** Data curation. **Song Yang:** Supervision, Resources. **Jian Jiang:** Writing – review & editing, Supervision, Resources, Funding acquisition, Conceptualization.

## Data and code availability statement

Data included in the article/supplementary material is referenced in the article.

## Declaration of competing interest

The authors declare the following financial interests/personal relationships which may be considered as potential competing interests:Jian jiang reports financial support was provided by 10.13039/501100010816Anhui Provincial Department of Science and Technology. Jingjing du reports financial support was provided by Huaibei Science and Technology Bureau. If there are other authors, they declare that they have no known competing financial interests or personal relationships that could have appeared to influence the work reported in this paper.
